# Supplementary material for: Association between in-stent neointimal characteristics and native coronary artery disease progression
Source: PLoS One. 2021 Apr 23;16(4):e0247359. doi: 10.1371/journal.pone.0247359 (PMC8064742; doi:10.1371/journal.pone.0247359)
Supplement: S1 Table — (DOCX) [file pone.0247359.s001.docx]

S1 Table. Quantitative coronary analysis of non-stented lesions occurred a non-target lesion revascularization at time of OCT evaluation

|  | Homogeneous  neointima  n= 18 (48.6%) | Heterogeneous  neointima  n= 13 (35.1%) | Layered  neointima  n= 6 (16.2%) | p-value |
| --- | --- | --- | --- | --- |
| Proximal reference vessel diameter, mm | 3.01 ±0.51 | 2.73±0.54 | 2.85±0.42 | 0.337 |
| Distal reference vessel diameter, mm | 2.72 ±0.60 | 2.51±0.53 | 2.34±0.30 | 0.292 |
| Minimal lumen diameter, mm | 1.67 ±0.48 | 1.50±0.53 | 1.40±0.28 | 0.421 |
| Diameter stenosis, % | 44.66 ±13.26 | 44.08±21.49 | 50.87±6.02 | 0.668 |
| Lesion length, mm | 15.88 ±7.96 | 13.50±3.79 | 16.76±8.08 | 0.527 |
| Mean duration from OCT to non-TLR, months | 60.89 ± 30.56 | 32.92 ± 26.38 | 43.33 ± 33.96 | 0.043 |

Data are given as mean ± SD, Non-TLR, non-target lesion revascularization; OCT, optical coherence tomography.
