# Supplementary material for: Association between in-stent neointimal characteristics and native coronary artery disease progression
Source: PLoS One. 2021 Apr 23;16(4):e0247359. doi: 10.1371/journal.pone.0247359 (PMC8064742; doi:10.1371/journal.pone.0247359)
Supplement: S2 Table — (DOCX) [file pone.0247359.s002.docx]

S2 Table. Subgroup analysis according to the reason for follow-up angiography.

|  | Evidence of ischemia  n=84 (22.3%) | Routine follow-up  n=293 (77.7%) | p-value |
| --- | --- | --- | --- |
| DCB at OCT evaluation | 59 (70.2%) | 21 (7.2%) | <0.001 |
| Homogeneous neointima | 32 (38.1%) | 175 (59.7%) | <0.001 |
| Heterogeneous neointima | 28 (33.3%) | 65 (22.2%) | 0.028 |
| Layered neointima | 24 (28.6%) | 53 (18.1%) | 0.028 |
| Non-TLR | 11 (13.1%) | 26 (8.9%) | 0.564 |
| Cardiac death | 1 (1.2%) | 4 (1.4%) | 0.874 |
| Any MI | 4 (4.8%) | 2 (0.7%) | 0.040 |
| TLR | 16 (19.0%) | 16 (15.5%) | <0.001 |
| Any revascularization | 24 (28.6%) | 36 (12.3%) | 0.003 |

Data are presented as number (%). DCB, drug-coated balloon; MI, myocardial infarction; Non-TLR, non-target lesion revascularization; OCT, optical coherence tomography; TLR, target lesion revascularization
