# Supplementary material for: Association between in-stent neointimal characteristics and native coronary artery disease progression
Source: PLoS One. 2021 Apr 23;16(4):e0247359. doi: 10.1371/journal.pone.0247359 (PMC8064742; doi:10.1371/journal.pone.0247359)
Supplement: S3 Table — (DOCX) [file pone.0247359.s003.docx]

S3 Table. Independent predictors for heterogeneous neointima

| Variable | Univariate analysis | | | Multivariate analysis | | |
| --- | --- | --- | --- | --- | --- | --- |
|  | OR | 95% CI | p-value | OR | 95% CI | p-value |
| Age | 1.018 | 0.992–1.044 | 0.181 | 1.014 | 0.988-1.042 | 0.292 |
| Time from PCI to OCT | 1.000 | 1.000-1.000 | 0.415 |  |  |  |
| Male | 0.912 | 0.552–1.508 | 0.720 | 0.993 | 0.585-1.686 | 0.979 |
| Diabetes | 1.111 | 0.682–1.810 | 0.673 |  |  |  |
| Hypertension | 0.849 | 0.526–1.370 | 0.503 |  |  |  |
| Dyslipidemia | 0.668 | 0.414–1.077 | 0.098 | 0.731 | 0.445-1.199 | 0.214 |
| Current smoker | 1.107 | 0.641–1.912 | 0.715 |  |  |  |
| CKD | 4.700 | 0.773–28.572 | 0.093 | 2.972 | 0.435-20.295 | 0.267 |
| MI at the time of OCT | 8.011 | 1.528–42.016 | 0.014 | 6.698 | 1.212-37.022 | 0.029 |
| First-generation DES | 0.860 | 0.520–1.421 | 0.556 |  |  |  |
| ACE inhibitor or ARB | 0.943 | 0.568–1.565 | 0.819 |  |  |  |
| Beta blocker medication | 0.591 | 0.344–1.017 | 0.058 | 0.631 | 0.359–1.110 | 0.110 |
| Statin | 0.941 | 0.424–2.087 | 0.880 |  |  |  |

ACEi, angiotensin-converting enzyme inhibitor; ARB, angiotensin receptor antagonist; CI, confidence interval; CKD, chronic kidney disease; DES, drug-eluting stent; MI, myocardial infarction; OCT, optical coherence tomography; OR, odd ratio; PCI, percutaneous coronary intervention.
